# The Association between Future Anxiety, Health Literacy and the Perception of the COVID-19 Pandemic: A Cross-Sectional Study

**DOI:** 10.3390/healthcare9010043

**Published:** 2021-01-05

**Authors:** Mariusz Duplaga, Marcin Grysztar

**Affiliations:** Department of Health Promotion and e-Health, Institute of Public Health, Faculty of Health Sciences, Jagiellonian University Medical College, Skawińska Str. 8, 31-066 Krakow, Poland; marcin.grysztar@uj.edu.pl

**Keywords:** future anxiety, COVID-19, conspiracy belief, health literacy, e‑health literacy

## Abstract

Increased anxiety related to the Coronavirus Disease 2019 (COVID-19) pandemic in society and specific professional groups has been reported by many authors. Most have applied tools enabling assessing the general traits of anxiety. Tools specifically designed for an assessment of anxiety or fear related to COVID-19 have also been developed. However, no study has assessed the future anxiety in relation to the pandemic. This concept was defined by Zaleski in the end of the 20th century as the state of apprehension, fear, worry, and concern regarding unfavourable changes in the more remote personal future. The aim of this study was an analysis to establish the level and the determinants of future anxiety in Polish society related to the COVID-19 pandemic three months after the introduction of the state of epidemic. The analysis reported in the paper is based on the data obtained through a web-based survey carried out on a representative sample of 1002 Polish adults aged 18–74 years. The hierarchical linear regression model was developed for the analysis of the determinants of future anxiety from the responses to a questionnaire consisting of five items. The independent variables selected for inclusion in the model, apart from sociodemographic characteristics, encompassed health literacy (HL) and ehealth literacy (eHL), perceived health threat related to COVID-19 (PHTC19), and a COVID-19-related conspiracy belief score (CCBS) derived from three items asking about the most popular conspiracy theories. The regression model developed in the final step showed that the future anxiety scale score (FASS) was significantly associated with gender, vocational status, HL, PHTC19, and CCBS. The FASS was lower among men than women (regression coefficient (B) (standard error, SE) = −1.28 (0.39), *p* = 0.001), among entrepreneurs or farmers rather than among employees of the public or private sector (B(SE) = −1.55, *p* = 0.010), in persons with a higher HL (B(SE) = −0.43 (0.06), *p* < 0.001). A higher FASS was observed in respondents with higher rather than lower PHTC19 (B(SE) = 1.49 (0.17), *p* < 0.001) and in those with a higher CCBS (B(SE) = 0.33 (0.07), *p* < 0.001). The model accounted for 15.2% of the variance of the FASS. In conclusion, the COVID-19 pandemic is not only a cause of increased mental symptoms, but also of increased future anxiety. Health-related measures are significantly associated with the FASS.

## 1. Introduction

The American Psychological Association defines anxiety as an emotion which is characterised by a feeling of tension, worried thoughts, and physical changes, e.g., tachycardia or increased blood pressure [1]. Anxiety can be a natural response to stress, however, for some people, it is a prominent, persistent, and disruptive element in their daily life [2]. Distinguishing between anxiety and anxiety disorders may be a challenge. It is often advocated that anxiety is an important adaptive mechanism signalling the need for a form of self-protective action to ensure one’s safety. Anxiety disorders, however, are characterised by recurring intrusive thoughts or concerns. Consequently, those suffering from anxiety disorders may avoid certain situations or activities. In the 1970s, the concept of anxiety being distinguished as either ‘state’ or ‘trait’ was introduced [3]. According to this concept, anxiety may be perceived as two complementary concepts: state anxiety identified as a psychophysiological state, or trait anxiety being a personality trait. State anxiety is the result of the individual’s psychological reactions directly related to adverse events, whereas trait anxiety is related to a personal inclination to exhibit anxiety. Such an understanding of the nature of anxiety entails the relative stability of trait anxiety over time. It also assumes that persons suffering from anxiety disorders would have higher trait anxiety than healthy people [4].

Anxiety may be experienced by individuals as a normal emotional reaction induced by known or unknown causes [5]. It may be a normal reaction to stress and could be perceived as the means of coping with it. An inherent element of anxiety is the anticipation of future dangers and a reaction which may help to avoid them. An anxiety response is triggered by psychological threats, unexpected or new situations and a person’s cognitive mechanisms. It may be also associated with specific medical conditions or the use of some substances, e.g., the excessive intake of caffeine, or even abstaining from others, e.g., alcohol. In its extreme forms anxiety may become a pathologic mechanism which no longer helps in coping with stress and difficult situations. Anxiety disorders are now the most common psychiatric diseases. According to estimates, nearly 30% of adults experience some form of anxiety disorder in their lifetime [6]. Anxiety disorders comprise an array of conditions, including panic disorders, phobias, obsessive-compulsive disorders, posttraumatic stress disorder, and generalized anxiety disorder [7]. It is believed that anxiety disorders result from an interaction between various biopsychosocial factors, such as a genetic susceptibility, which interacts with stress or traumatic situations. The importance of interactions between the genetic background and the environment is supported by the individual differences in coping with stress and the occurrence of anxiety disorders [6].

The Coronavirus Disease 2019 (COVID-19) pandemic has triggered many studies focused on assessing the prevalence of anxiety in both the general population and in specific social groups, e.g., healthcare professionals. A study performed in Spain during the initial phase of the epidemic revealed that anxiety was prevalent in 26% of women and 14% of men in the general population (26% vs. 14%) [8]. The study carried out by Hyland et al. in Ireland, revealed that 20% of respondents exhibited symptoms of a general anxiety disorder and 22.8% exhibited symptoms of depression [9]. A study carried out in Germany revealed that, in the general population, 45% were experiencing increased generalised anxiety, 59% had a COVID‑19-related fear, 65% showed psychological distress and 14% were in a state of depression [10]. According to a study performed in Iran, anxiety occurred more frequently in respondents living in the areas of high COVID-19 prevalence than in those living in areas with lower prevalence, and in persons having a family member, relative or friend who had contracted COVID-19, among women rather than men, among persons with a University level of education than a lower level, and among those who have been more active in following coronavirus-related news items [11]. A systematic review prepared by Salari et al. [12] showed that during the COVID-19 pandemic, stress was experienced by nearly 30%, anxiety by 32% and depression by 34% of the general population. These reports indicate that the COVID-19 pandemic is associated with an increased prevalence of anxiety. A systematic review published by Baxter et al. in 2013 showed that the global prevalence of anxiety disorders, adjusted for methodological differences was 7.3% [13]. The adjusted prevalence varied from 5.3% for Indo/Asian and African cultures to 10.4% for European/Anglo cultures. According to the Our World in Data report, anxiety disorders are experienced, depending on the country, by 2.5–7.0% and depression by 2.0–6.0% of their general population [14]. Valid comparisons with the results of pre-pandemic studies and those undertaken during the pandemic are not totally reliable because of differences in the study designs and the applied tools, but a general assessment seems to support the view that the COVID-19 pandemic is related to an increased prevalence of anxiety in the general population.

There are also many reports on the prevalence of anxiety among health care professionals. According to Teng et al. during the COVID-19 pandemic in China anxiety was observed in 23.4% and moderate to severe anxiety in 7.5% of the frontline staff [15]. Hacimusalar et al. reported that the levels of anxiety and hopelessness during COVID-19 pandemic were higher among health care workers in Turkey than in other professional groups [16]. Increased working hours was one of the most significant factors associated with increased anxiety. Furthermore, the level of anxiety among nurses was higher than that in other health care workers. Cao et al. assessed the psychological consequences of COVID-19 pandemic in more than 7000 medical students in Chanzhi, China [17]. The analysis based on the Generalized Anxiety Disorder Scale (GAD-7) revealed that 21.3% respondents experienced mild, 2.7% moderate and 0.9% severe anxiety. The factors alleviating anxiety included residing in a rural area, a stable family income and living with parents. A higher level of anxiety was experienced by students whose relatives or friends had been infected with COVID-19. A systematic review performed by Pappa et al. showed that the pooled prevalence of anxiety among healthcare professionals was 23.2% and 22.8% that showed symptoms of depression [18].

A particularly high prevalence of anxiety and depression was observed among quarantined persons. According to Tang et al., for those in quarantine, the prevalence of symptoms of anxiety could be up to 71.0% and of depression 26.5% [19]. The prevalence of mental health symptoms in patients with COVID-19 disease is outside the scope of this paper. However, it should be underlined that high level of psychiatric symptoms were reported among COVID-19 survivors. For example, Mazza et al. found that 55% of survivors may suffer from at least one mental disorder [20]. In the prospective cohort study carried out in San Raffaele Hospital in Milan, anxiety was reported in 42% and depression in 31% of the sample of 402 adults. Various tools have been applied for the assessment of the anxiety in the studies carried out during the COVID-19 pandemic. Most frequently the researchers have used the GAD-7 [10,17,19], the Depression Anxiety and Stress Scales (DASS-21) [8,11], the Self-Rating Anxiety Scale (SAS) [15] and the State-Trait Anxiety Inventory (STAI) [16].

Some authors developed COVID-19-specific tools to assess anxiety or fear related to the current pandemic. Lee developed a five-item, Coronavirus Anxiety Scale (CAS) as a brief mental health screener to diagnose cases of dysfunctional anxiety related to the COVID-19 pandemic [21]. According to this author, in the sample of 775 adults from the USA a CAS score was associated with the diagnosis of coronavirus infection, or the use of alcohol and drugs, negative religious coping, extreme hopelessness, and passive suicidal ideation. Furthermore, higher scores were found in Asians than in white and black populations, among younger rather than older people, and among those with higher rather than lower levels of education. Interestingly, the CAS score was positively correlated with the approval of President’s Trump response to the coronavirus pandemic and the intention to boycott Chinese food and products in the future. Lee did not find a relationship between CAS score and gender or a history of anxiety. The Fear of COVID-19 Scale (FC19S) is another brief tool developed by an international team of researchers [22]. A validation demonstrated its robust psychometric properties and correlation with the results of the assessments using other related tools including the Hospital Anxiety and Depression Scale and the Perceived Vulnerability to Disease Scale. Currently, several validated linguistic versions of the FC19S are available [23,24,25,26,27,28,29].

A person’s economic circumstances are an important factor influencing the general level of anxiety during the pandemic. Mann et al. assessed the level of personal economic anxiety in a sample of approximately 500 adults from the USA [30]. The study, using an adapted version of the economic hardship scale, revealed that only 15% of participants reported no or low economic anxiety [31]. The authors found that lower economic anxiety was shown by older persons, white respondents, the retired or disabled, and those earning more than $75,000 a year. However, higher economic anxiety was revealed by those who had children living at home.

In most studies, the prevalence of anxiety was assessed together with fear, stress or depression. The potential predictors assessed in these studies were most frequently the sociodemographic variables and features related to the course of the COVID-19 pandemic. The association between health literacy (HL) or e‑health literacy (eHL) and anxiety or fear in general population was studied less frequently. HL and eHL are concepts directly related to the understanding and use of health information. HL was defined in 1998 by the WHO as “the cognitive and social skills which determine the motivation and ability of individuals to gain access, understand and use information in ways which promote and maintain good health” [32]. According to the model developed during the European Health Literacy Survey Project, HL may be perceived as a matrix of 12 dimensions resulting from the combination of four types of activities related to health information and the three domains: healthcare, disease prevention and health promotion [33]. The available evidence indicates that adequate HL is a precondition for patient empowerment and the efficient use of health care resources, effective communication with health care providers and the daily implementation of the rules for a healthy lifestyle [34,35,36,37,38]. eHL, also called digital health literacy, is a term used in parallel with HL in relation to health information available from the Internet and other electronic sources. In 2006, Norman and Skinner proposed a definition and a model of eHL originating from a set of six basic competencies including reading and calculating, as well as literacies relevant to health, information, science and information technology [39]. eHL encompasses the abilities to search for, access, understand and appraise health-related information obtained from electronic resources and to use it for solving health-related problems. It is assumed that eHL should protect people from the consequences of accessing unreliable health information on the Internet. The assessment of eHL may be particularly important for the matching of eHL to skills and the abilities of potential users, patients and the general population. Many authors have postulated that both HL and eHL may be particularly important during the current pandemic regarding the adherence to recommended preventive measures and resilience to the co-existing infodemic, the spreading of conspiracy beliefs and the resulting fear and anxiety [40,41,42,43,44,45,46,47]. The relative scarcity of studies analysing the association of HL and eHL with the consequences of the pandemic is somewhat surprising and even those already published present ambiguous results. The study undertaken on adults with chronic conditions in the USA showed that lower worries about COVID‑19, a lower perception of the risk of the infection, and a lower self-assessed preparedness for an epidemic were demonstrated by persons living in poverty, and by those possessing a low level of HL [48]. In turn, the study carried out among medical students from Universities in Vietnam revealed that a higher HL was associated with a lower score on the scale used for assessing fear related to COVID-19 [49]. Another study in Vietnam, on persons with suspected COVID-19, demonstrated that higher HL was related with a lower likelihood of suffering from depression and a higher level of a health-related quality of life [50].

The COVID-19 pandemic has revealed that the great public health challenges are accompanied and impeded by various forms of misinformation [51]. In February 2020, the World Health Organisation introduced the term infodemic to describe the flood of fake news, conspiracy theories and manipulated information accompanying the COVID-19 pandemic [52]. Conspiracy theories reject the standard explanation of an event and attribute it to covert groups or organisations intending to carry out secret plots. Both psychological stress and anxiety have been postulated as factors related to belief in conspiracy theories. According to Hofstadter this may result from the fact that belief in a conspiracy theory offers a simplified explanation of stressful events and facilitates a feeling of regaining control in situation associated with acute stress [53]. The association between fear, anxiety, and conspiracy beliefs was reported by Grzesiak-Feldman [54]. The study performed by Swami et al. revealed that more stressful life events and greater perceived stress were associated with a belief in conspiracy theories [55], however a similar association has not been confirmed for state or trait anxiety. Green and Douglas observed that there is a significant association between anxious attachment and belief in conspiracy beliefs [56]. Significantly, recent studies carried out during the COVID-19 pandemic indicate that conspiracy beliefs are associated with higher anxiety [57,58]. The COVID-19 pandemic is perceived by many individuals as a threat to their future and that of societies. The resulting increased future anxiety may be related not only to health-related threats but also to the menace of potential economic and political instability. The concept of future anxiety was introduced by Zaleski in 1996 [59] and it was suggested that various forms of anxiety have some relationship with the anticipated future. However, in the concept of future anxiety, future corresponds with greater temporal distance. He defined it as a state of apprehension, uncertainty, fear, worry and concern that unfavourable changes are likely in the more remote personal future. In extreme cases, a person’s future anxiety could be combined with the conviction that something catastrophic may happen to them. The feelings combined in the future anxiety may reflect the uselessness of a person making efforts to achieve a desired state. According to Zaleski, future anxiety has a strong cognitive and limited physiological component [59]. He refers to the cognitive concepts of anxiety proposed by other authors, particularly Eysenck [60]. Eysenck proposed a hypervigilance conception which assumed that the cognitive approach to anxiety stems from an assumption that the major function of anxiety is to enable threat or impending danger to be detected. Zaleski et al. developed the Future Anxiety Scale (FAS) as the individual’s self-reported measure of anxiety related to the perception of their future. Five FAS questionnaires have been developed, FAS1 to FAS5, each having a different number of items, ranging from 56 in FAS2 to only 5 in FAS5. The five-item version (FAS5) has also been called the Dark Future Scale (DFS) [61]. A positive correlation was shown between DFS and the Future Negative Scale, the subscales of Zimbardo Time Perspective Inventory (past-negative, present-hedonistic, present-fatalistic and future) as well as with the Carpe Diem Scale [61]. Previous studies confirmed that higher future anxiety was associated with manipulative treatment of others [62] and higher pessimism when predicting solutions to global problems [63]. Bujnowska reported also that the parents of children with disabilities exhibited greater future anxiety than those having healthy children [64].

The first case of the SARS-CoV-2 infection in Poland was confirmed with a laboratory polymerase chain reaction (PCR) test on 4 March 2020 [65]. Then, on 10 March a local transmission phase of COVID-19 in the country was announced [66]. On 10–12 March, lockdown measures including cancelling mass events, the initial closure of schools and universities, followed by the introduction of remote teaching activities and strict regulations on gatherings were imposed. The lockdown rules were intensified on 31 March with the requirement for social distancing in public places, the restricted access to parks, promenades and using services based on direct person to person contact, e.g., hairdressers. Unaccompanied minors were not permitted to leave homes. In June 2020 and later during the summer holiday season, the lockdown restrictions were reduced, or at least not so strictly enforced as earlier. In March 2020 the total number of new cases of COVID-19 was 2311, in April there were 10,566 and in May 10,909. From the beginning of June to the end of August, the daily number of new reported cases ranged from 230 to 900. By 31 August 2020 the total number of COVID-19 cases in Poland was 67,372, which has resulted in 2039 deaths. In many other countries, the numbers of new cases of COVID-19 registered until the end of August 2020 were proportionally much higher [67]. After children returned to schools in September, a surge in morbidity and mortality occurred, with 25,221 new cases and 430 deaths due to COVID-19 being reported on 11 November 2020 [67].

This paper reports on the assessment of future anxiety in the Polish population following the first phase of the COVID-19 pandemic lockdown measured with the DFS developed by Zaleski et al. An analysis of the association of the future anxiety with HL, eHL, the perceived health threat and the level of conspiracy beliefs related to COVID-19 was undertaken. The study was carried out in mid-June, a period when relatively low numbers of new cases of COVID-19 were being reported daily, i.e., less than 600 per day. In comparison to other countries, the burden of the pandemic in Poland at this time was relatively low.

## 2. Materials and Methods

### 2.1. Survey

The data used in this study were obtained from a computer-assisted web-based interviewing (CAWI) survey on a representative sample (N = 1002) of Polish adults’ population aged 18–74. The survey was undertaken in mid-June 2020 by the PBS Company, which is widely experienced in carrying out opinion polls [68]. It adheres to the quality programme issued by the Polish Association of Public Opinion and Marketing Research (Organizacja Firm Badania Opinii i Rynku) [69]. The PBS Company maintains an Internet research panel in line with the requirements of legislation on personal data protection. The study sample was adjusted for age, place of residence, the level of education and NUTS1 regions. The allocations of respondents to a specific group were derived from the data provided by the main statistical office in Poland [70].

The survey was carried out after being approved by the Bioethical Committee of the Jagiellonian University in Krakow (No 1072.6120.99.2020 of 23 April 2020). The questionnaire was completed anonymously by the respondents after receiving an explanation of the objectives of the study and giving their consent to participate in the study.

### 2.2. Questionnaire

The analysis was based on the questionnaire consisting of 55 items. The short, 16-item version of the European Health Literacy Survey Questionnaire (HLS-EU-Q16) was used to assess HL [71] and the Polish version of the eHealth Literacy Scale (Pl-eHEALS) to determine the respondents’ eHL [72]. The Internal reliability of the instruments, Cronbach α, 0.90 and 0.89, respectively, was appropriate. The level of belief in conspiracy theories was measured by three items asking about the popular conspiracy theories circulating during the COVID-19 pandemic. An earlier assessment of this ad-hoc tool showed an acceptable internal reliability (Cronbach’s α = 0.73) [73]. The future anxiety was measured with a brief, 5-item tool developed by Zaleski et al. [61]. The internal reliability of the tool was satisfactory (Cronbach’s α = 0.89). The questionnaire included also of a set of questions about COVID-19-related information behaviours and attitudes as well as sociodemographic items. The order of presentation of specific items in the online questionnaire was as follows: the HLS-EU-Q16, the Pl-eHEALS, questions asking about perceptions about the COVID‑19 pandemic, the DFS and a set of items asking about belief in conspiracy theories.

### 2.3. Measures

The HL score was calculated using the method recommended by the European Health Literacy Survey project team [71] and has been applied in subsequent Polish studies [38]. The response options, “easy” and “very easy”, were assigned with the value “0” and response options “difficult” and “impossible” with the value “1”. The response “difficult to say” was assumed to be a missing value. Total HL score was calculated if the number of missing values was lower than 2 by summing individual scores. The eHL score was determined in line with the instructions given by the authors of the eHealth Literacy Scale (eHEALS) [74]. It is calculated as a sum of individual scores obtained after assigning values of 1 to 5 to the responses from “I decidedly do not agree” to “I decidedly agree”. A Polish version of the scale was validated earlier [72]. The COVID-19-related Conspiracy Beliefs Score (CCBS) was the sum of the individual responses to three relevant items asking about believing conspiracy theory given values of 1 to 5, where ‘I decidedly agree’ was assigned the value of 5 to ‘I decidedly do not agree’ a value of 1 [73]. The perceived threat to health arising from the COVID-19 pandemic (PHTC19) for each respondent and his/her family was assessed with one question to which response could be given a value according to the 5-item Likert scale, from ‘I decidedly do not agree’ 1 to ‘I decidedly agree’ 5. In turn, the FAS Score (FASS) was calculated as the sum of individual responses to 5 items included in the DFS. The individual responses could assume the values from 1 to 7 (with ‘decidedly true’ given the value 7 and “decidedly wrong” the value 1) [61].

### 2.4. Statistical Analysis

The statistical analysis was performed using the IBM SPSS v.24 software (IBM Corp., Armonk, NY, USA). For the categorical variables absolute and relative frequencies were provided and for numerical variables—the mean and standard deviation.

In the first step of regression analysis, the univariate linear models were used to assess the relationships between the FASS and sociodemographic variables, HL, eHL, the PHTC19 and the CCBS. Then, a hierarchical regression model consisting of 4 stages was followed. Only the independent variables for which p reached at least the value 0.1 in the univariate models, were included in consecutive stages of the hierarchical modelling. At every stage, the ANOVA test for the model was calculated as well as the F statistics for the changes of the R^2^ coefficients. Unstandardised regression coefficients (B), standard errors (SE), standardised regression coefficients (β), 95% confidence intervals (95%CI), and p values were provided for the independent variables used in the linear regression models. Only *p* values < 0.05 were deemed to be significant. The *p* values lower than 0.01 were reported to three decimal places, otherwise to only two decimal places.

## 3. Results

### 3.1. Characteristics of the Study Group

The study sample was representative of the population of Polish adult users of the Internet of which 50.6% were women. A University degree was possessed by 31.2%. There were 36.6% of the sample inhabiting rural areas and 22.3% living in cities with a population of at least 200,000. The proportion of married persons was 50.8% and singles 34.5%. Public or private sectors employees made up 47.2% of the group, self-employed or farmers 13.7%, University or school students 10.2% and retired or on disability pension 9.6%. The detailed characteristics of the study group was published earlier [73].

Mean (standard deviation, SD) values of the scores measured in the study were as follows: HL—12.87 (3.42), eHL—29.74 (5.14), PHTC19—3.28 (1.22), CCBS—10.25 (2.78), and FASS—22.30 (6.44). The distribution of responses to individual items of the DFS is shown in Table 1. The correlation matrix for interval variables used in regression analysis is shown in Table 2.

### 3.2. Univariate Analysis of Future Anxiety

**Figure 1:**
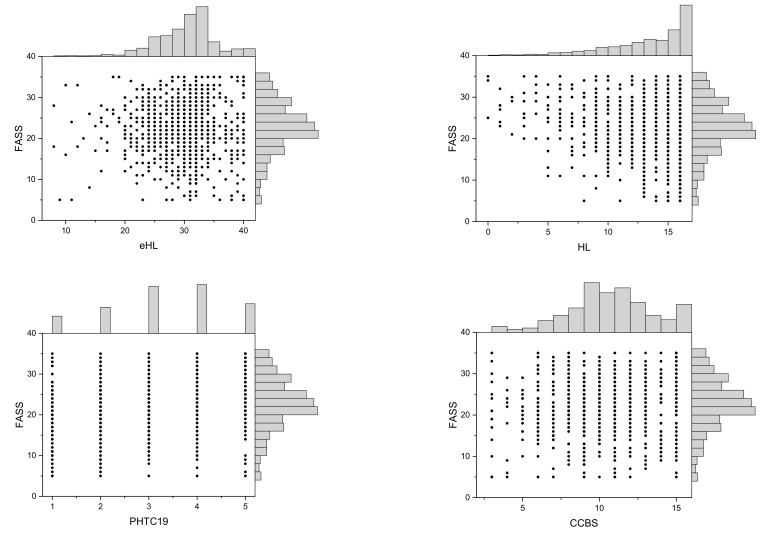
Scatterplots with marginal histograms for FASS and selected independent variables (abbreviations: FASS—future anxiety scale score, HL—health literacy, eHL—ehealth literacy, PHTC19—the perceived health threat related to the COVID-19 pandemic; CCBS—COVID-19-related conspiracy belief scale.

The univariate linear regression revealed that the FASS was associated with gender, income, vocational status, HL, PHTC19 and CCBS. A lower level of future anxiety was found among men than among women (B = −1.62; *p* < 0.001). Persons living in a household with a monthly net income per inhabitant <1500 Polish zlotys (PLN) had a higher level of future anxiety than those living in household with an income 1500–3000 PLN (B = 0.99, *p* = 0.048). Furthermore, the level of future anxiety was significantly lower for the self-employed or farmers than for the public or private sector employees (B = −1.56, *p* = 0.012). Higher HL was also associated with lower future anxiety (B = −0.43, *p* < 0.001). Higher PHTC and higher CCBS score were associated with a higher future anxiety (B = 1.32, *p* < 0.001 and B = 0.19, *p* = 0.010, respectively). eHL was not associated with the level of future anxiety (B = 0.001, *p* = 0.97, R^2^ < 0.001). The details of the analysis are given in Table 3. The scatterplots with marginal histograms for FASS and interval predictor variables are shown in Figure 1.

### 3.3. Hierarchical Linear Regression Modelling of Future Anxiety

The hierarchical linear regression consisted of four stages. In the first stage, sociodemographic variables were introduced to the FASS model. Of the initial three variables only two retained a significant association with the FASS. The resulting model explained 2.3% of the variance of the score. A lower level of future anxiety was shown by men than women (B = −1.43, *p* = 0.001) and entrepreneurs or farmers compared to employees (B = −1.66, *p* = 0.010). In the second step, the HL score was added to the model. It explained an additional 5.4% of the variance of the FASS. In the third step, the variable reflecting the PHTC19 was included and the corrected R^2^ was increased by a further 5.7%. In the final step, the CCBS score was included resulting in an increase of R^2^ by 1.8%. All the changes of R^2^ were statistically significant. To sum up, the model including sociodemographic variables, HL, PHTC, and CCBS explained 15.2% of the variance of the FASS. In this model, the independent variables which were associated significantly with the FASS included gender, vocational status, HL, PHTC19, and CCBS. The details of the analysis are shown in Table 4.

## 4. Discussion

The purpose of the analysis undertaken in this study was to determine the association between future anxiety and HL, eHL, and PHTC after making adjustment for sociodemographic factors. The final model obtained with hierarchical linear regression approach explained 15.2% of the variance of the FASS. From the sociodemographic variables, only gender and vocational status maintained a significant effect on FA. In addition, it was statistically associated with HL but not eHL. Persons possessing a higher HL experienced lower level of FA. The PHTC19 was a significant component of the model explaining an additional 5.7% of variance in the FASS. A higher perceived threat was related to a higher FASS. The CCBS, based on items asking for an opinion on three of the most widely circulating conspiracy theories was also significantly associated with FASS. Persons expressing a stronger belief in conspiracy theories suffered from a higher FA. It should be emphasized that the variables reflecting health-related attitudes and competencies were able to explain a relatively small part of the variance in the FASS. It is clear that other factors play a significant role in determining the level of the anxiety related to the perception of the future during the pandemic.

Increased anxiety, apart from fear, symptoms of depression, and lower quality of sleep, was the most frequently reported mental consequence of the COVID-19 pandemic in the general population [75,76,77]. Similar findings were confirmed for health care professionals [78] and patients who had suffered from the symptoms of COVID-19 infection [79]. A better understanding of the determinants of anxiety during COVID-19 pandemic is of the utmost importance as symptoms of anxiety and depression may result in people being less likely to adhere to the recommended preventative measures [80]. It may also provide evidence supporting changes in mental health care provision as postulated in a position paper by Moreno et al. [81].

In this study, of the sociodemographic variables, only gender and vocational status were associated with the level of FA. To date, no study carried out during the COVID-19 pandemic has focused on the FA assessed using the tool proposed by Zaleski et al. In studies based on other tools measuring the general level of anxiety, it was found that women consistently displayed a higher level than men [8,11,82,83,84,85,86]. Interestingly, the level of future anxiety in the Polish population was not associated with the place of residence, marital status or the level of education. Similar findings were reported by Wong et al. [83], while in other studies greater anxiety was found in persons possessing a higher level of education ([11,86] and among married persons [82]. In the American study performed by Lee [21], the level of the COVID-19-specific anxiety depended on race, age, level of education and views on political aspects in the USA. A significant association between the level of anxiety and age, apart from gender, was also reported in the general population of Iran [85].

In Ecuador in patients remaining under surveillance because of COVID-19, the degree of anxiety symptoms measured with GAD-7 was significantly associated with gender and their behaviours during the period of confinement [87]. Men, those keeping a regular daily schedule, those continuing to take physical exercise, and those spending an hour or less seeking information about COVID-19, compared to those avoiding the topic, or spending more time searching for information, had lower GAD-7 scores. The protective effect of physical exercise in relation to the prevalence of symptoms of anxiety during the COVID-19 pandemic was also confirmed in inhabitants of Wuhan, China [82].

It may be surprising that potential effects of HL in relation to COVID-19, Severe Acute Respiratory Syndrome (SARS), and Middle East Respiratory Syndrome (MERS) epidemics have not been subjected to detailed studies. A systematic review prepared by Seng et al. showed that not one of 70 papers included in their analysis evaluated the outcomes associated with HL during epidemics caused by the new, emerging types of coronaviruses. However, researchers had frequently assessed people’s knowledge about infections, the resulting worries and the use of preventive measures [88]. After the article by Seng et al. was posted online as a preprint on 11 May 2020, more evidence has emerged. For example, Riiser et al. showed that, among adolescents in Norway, HL was positively associated with the knowledge about the need for handwashing and their behaviour [89].

According to the analysis of responses of adult Internet users in Poland, a higher HL is associated with a lower level of future anxiety. This finding is in line with the results of studies carried out in other countries. According to McCaffery et al., using the STAI questionnaire on a sample of adult Australians, an inadequate level of HL was associated with a lower perceived seriousness of threat, but a higher level of anxiety [90]. Wolf et al. also found a greater degree of anxiety in patients living in the USA suffering from chronic diseases who also had low HL [48]. A higher HL was reported to give protection against the occurrence of symptoms of depression in people suspected of having COVID‑19 [50] and likewise for medical students the fear related to COVID-19 [49].

There was no association between eHL and FA in the sample of Polish respondents. Contrary to the expectations expressed by some authors, eHL seems to have no added value in counteracting the potential negative consequences of the pandemic, at least for those related to increased stress and anxiety. Theoretically, persons with higher eHL should have the ability to efficiently handle the flood of health-related information available on the Internet. The analysis presented in this paper was based on the Polish version of eHealth Literacy Scale [72], the original version being introduced initially by Norman and Skinner in 2006 [74]. It consists of items examining the self-perceived ability to find, appraise and apply the health-related information available on the Internet. Providing that most Internet users also explore health-related resources available online, one could expect that a higher eHL measured with eHEALS, would be related to a lower level of anxiety, particularly as high exposure to the news presented in various media during a pandemic may be associated with higher levels of anxiety. Nourisaeed et al. reported that being overburdened with online health-related information may lead to higher COVID-19-related anxiety [91]. Similar results were reported by other authors [10,92]. In addition, Sigurvinsdottir et al. reported that information seeking may be associated with more symptoms of depression, anxiety or stress [93].

The findings described here seem to be in agreement with the observations of Lee et al. [94]. They did not find a statistically significant relationship between eHL and the level of anxiety, neither among older persons from the USA nor from South Korea [94]. It should be added that an earlier study performed in a representative sample of the Polish population suggested that attitudes to key public health interventions were related to the level of HL but not eHL (measured with Pl-eHEALS) [95]. Furthermore, eHL was not a significant predictor of the quality of telehealth experience during the COVID-19 pandemic in the sample of patients from Australia [96]. In agreement with the findings described in our paper, Kubb & Foran did not observe a meaningful contribution of eHL to a parent’s change of stress level when searching the web for current somatic health issues related to their own or their children’s symptoms [97]. However, there is one study reporting that eHL positively moderates the relationship between the frequency of using social media and preventive behaviours [98].

The study reported here revealed that a higher perceived threat related to COVID-19 was associated with greater level of future anxiety. This finding is in line with the observations made in Iran by Moghanibashi-Mansourieh [11], who reported a higher level of anxiety among persons who had family members, relatives or friends who contracted COVID-19 or were living in areas having high prevalence of the disease. Lin et al., using the STAI questionnaire, found that people in China who were convinced about a higher susceptibility, severity, and impact of COVID-19 showed a greater degree of anxiety [84]. In general, the assessment of fear or stress related to COVID-19 with specific tools, like the FC19S, showed that these scores correlated with the general anxiety scores [22,23,28,29,99]. Taylor et al. described five main facets of the concept of the COVID-19 stress syndrome [100]. These were the fear of the dangerousness of COVID-19; worries about the socioeconomic costs; fears that foreigners are spreading a new coronavirus; traumatic stress symptoms related to the direct or vicarious exposure to COVID-19 and finally, COVID-19-related compulsive checking and seeking reassurance. The survey carried out on a large sample of about 6850 American and Canadian adults, indicated that feeling of the threat resulting from COVID-19 was a central feature of the syndrome [100].

The positive association between conspiracy beliefs and the level of FA is fully in agreement with other studies. The conspiracy beliefs were assessed with an ad-hoc tool consisting of three items asking about respondents’ opinions on the most common conspiracy theories circulating in Poland. Sallam et al. observed that among students from Jordan, higher anxiety was associated with the belief that COVID-19 was the result of a global conspiracy [57]. The belief in conspiracy theories related to COVID-19 was also associated with a higher level of anxiety in the country’s general population [101]. According to Liu and Tong, the exposure to updates or rumours about COVID-19 was associated with increased anxiety [102]. The study performed by Srol et al. shortly after the first cases of COVID-19 were identified in Slovakia, revealed that a higher perception of risk and a lower trust in institutions in the context of the COVID-19 pandemic were associated with anxiety and the feeling of having no control [58]. Furthermore, they found that the relation between the perception of risk as a predictor and believing conspiracy beliefs about COVID-19 was mediated by the level of anxiety. Interestingly, those health care professionals in Ecuador who believed that a new coronavirus was developed intentionally in laboratory, also showed higher levels of distress and anxiety [103]. Other studies confirmed a significant relationship between beliefs in conspiracy theories related to COVID-19 and the level of anxiety and stress [104,105]. It should be noted that the significance of beliefs in conspiracy theories goes beyond an impact on people’s mental health during a pandemic. As demonstrated by Barua et al. conspiracy beliefs may have a negative impact on the individual’s responses to the recommended countermeasures the during COVID-19 pandemic [106]. Interestingly, the study of Farias and Pilati showed that the impact on preventive measures may be nuanced depending on the specific conspiracy theories being shared by respondents [107]. No association between COVID-19 related conspiracy beliefs and anxiety was found by Georgiou et al. among respondents from the USA and several European countries [108]. The authors postulated that their study was performed on a relatively young population with a lower perceived threat from the COVID-19 and the study was made in an early phase of lockdown in the most affected countries, which precluded detecting the effects of prolonged periods of isolation.

The variables significantly associated with general anxiety level in other studies, but not covered by the analysis reported in this paper, include trust in governmental actions to combat COVID-19 [10] and the subjective level of information regarding COVID-19 [10] or media consumption [92]. Interestingly, the higher subjective level of information was positively associated with COVID-19-related fear but negatively with generalised anxiety symptoms [10]. Nekliudov et al. reported that excessive exposure to media focusing of COVID-19 may be associated with increased anxiety [92]. Meyer et al. showed that increased screen time was associated with a higher intensity of depression symptoms, but not with the level of anxiety [109]. Holman et al. observed that there was a significant association between acute stress and depressive symptoms, and daily COVID-19-related media exposure and the conflicting information regarding COVID-19 in the media among respondents in the USA [110]. Increased anxiety measured with GAD-7 in persons demonstrating excessive media usage was confirmed in the UK population [111].

Grey et al. assessed the role of perceived social support in relation to mental symptoms occurring during the COVID-19 pandemic [112]. They did not find a significant association between the level of social capital and anxiety (measured with GAD-7) after adjustment for any potential confounders. However, a significant relationship was found for the scores of depression and sleep quality. The economic effects, the impact on daily life, and delays in academic activities caused by the COVID-19 pandemic were reported as being related to increased anxiety symptoms among college students in China [10].

### Limitations

Due to limitation on the size of the online questionnaire, not all items that could be relevant for an assessment of FA in the COVID-19 pandemic have been included in the analysis. In this study, the importance of factors related to the perceptions and skills related to the health context after adjusting for sociodemographic factors were analysed. The predictors included in the final step of the hierarchical linear regression accounted for about 15% of the FASS variance. Apparently, other variables, not related to the health context, also play an important role in the prevalence of anxiety during the pandemic.

It would be interesting to assess how an increased FA correlates with COVID-19-specific anxiety, fear or stress. Unfortunately, in May 2020, when the decision from the Bioethical Committee was sought, no tool had been adopted for Polish language. Among other limitations, the tool used to measure beliefs in COVID-19-related conspiracy theories was based on three questions only and potentially important areas of conspiracy may have been omitted. Further, it was an ad hoc tool based on an arbitrary selection of items.

## 5. Conclusions

The COVID-19 pandemic is not only causing threats to the health of citizens, but it is also the reason for significant disturbances in the social and economic situation of many countries. It is obvious that, from a short-term perspective, the pandemic has resulted in a substantial psychological burden on society and has increased the prevalence of psychiatric symptoms, including anxiety, depression, and stress. The perception of the resulting risks does not only involve health issues, but also raises questions about the economic and social stability of individuals, their families, and communities. Most research targeting an assessment of anxiety in the current circumstances applied tools measuring the general level of anxiety symptoms, and not future anxiety. In this study, the role of health-related competencies and attitudes in shaping the perception of future anxiety were analysed. After adjusting for sociodemographic factors, HL, the perceived health threat from COVID-19 and a belief in related conspiracy theories were found to have a significant association with the FA. These factors were responsible for a change of less than 15% of variance of the FASS. Unexpectedly, only HL but not eHL was found to have a significant effect on FASS using the hierarchical linear regression model. These findings seem to be counterintuitive and opposite to many postulates suggesting the important role of eHL in moderating individual responses to the flow of online information related to the pandemic. In future research, it would be interesting to determine the degree to which future anxiety acts as the motivation for individuals to become involved in preventative or anticipatory activities and, in the context of the COVID-19 pandemic, how it is related to the willingness to adopt measures aimed at disease containment.

## Figures and Tables

**Table 1:** The distribution of responses to the Dark Future Scale.

Item	Decidedly Wrong	Wrong	Rather Wrong	Difficult to Say	Rather True	True	Decidedly True
I am afraid that the problems which trouble me now will continue for a long time.	6.8 (68)	7.5 (75)	11.9 (119)	36.9 (370)	21.5 (215)	9.3 (93)	6.2 (62)
I am terrified by the thought that I might sometimes face life’s crises or difficulties.	6.1 (61)	6.8 (68)	11.6 (116)	25 (250)	26.2 (263)	14.9 (149)	9.5 (95)
I am afraid that in the future my life will change for the worse.	6.4 (64)	6.3 (63)	11.2 (112)	24.7 (247)	25.0 (250)	16.3 (163)	10.3 (103)
I am afraid that changes in the economic and political situation will threaten my future.	4.5 (45)	6 (60)	8.1 (81)	23.4 (234)	27.4 (275)	18.1 (181)	12.6 (126)
I am disturbed by the thought that in the future I won’t be able to realise my goals.	4.4 (44)	6.8 (68)	8.2 (82)	23.4 (234)	27.1 (272)	18.5 (185)	11.7 (117)

**Table 2:** Correlation matrix for selected measures analysed in regression models (Spearman ρ coefficients).

	FASS	HL	eHL	PHTC19
HL	−0.21 **			
eHL	0.01	0.40 **		
PHTC19	0.24 **	0.01	0.11 **	
CCBS	0.08 *	−0.03	0.10 *	−0.26 **

* 0.01 < *p* < 0.01, ** *p* < 0.001. Abbreviations: FASS—future anxiety scale score, HL—health literacy, eHL—ehealth literacy, PHTC19—the perceived health threat related to the COVID-19 pandemic; CCBS—COVID-19-related conspiracy belief scale.

**Table 3:** Univariate linear regression modelling for FASS.

	Variable	B	SE	β	LL	UL	*p*
sex	men vs. women	−1.62	0.40	−0.13	−2.41	−0.83	<0.001
age		0.00	0.01	0.00	−0.03	0.03	0.97
education	upper secondary or post-secondary non-tertiary	ref.					
	lower than upper secondary	0.07	0.54	0.00	−0.99	1.13	0.90
	Bachelor’s degree	1.16	0.69	0.06	−0.19	2.51	0.091
	Master’s degree or higher	0.14	0.53	0.01	−0.90	1.19	0.79
income	1.500–3.000 PLN	ref.					
	≤1.500 PLN	0.99	0.50	0.07	0.01	1.98	0.048
	>3.000 PLN	−0.51	0.57	−0.03	−1.63	0.61	0.37
	refused to disclose	−0.50	0.64	−0.03	−1.77	0.76	0.44
marital status	married	ref.					
	single	0.40	0.45	0.03	−0.48	1.28	0.37
	widowed or divorced or in separation	0.25	0.60	0.01	−0.93	1.43	0.68
vocational status	employee of public or private sector	ref.					
	self-employed or farmer	−1.56	0.62	−0.08	−2.78	−0.34	0.012
	on a disability pension or retired	−0.34	0.72	−0.02	−1.75	1.07	0.64
	University or school student	0.04	0.70	0.00	−1.34	1.42	0.95
	vocationally inactive incl. unemployed	0.75	0.55	0.05	−0.32	1.82	0.17
place of residence	rural	ref.					
	<20,000	0.13	0.70	0.01	−1.24	1.51	0.85
	20,000–200,000	0.25	0.50	0.02	−0.73	1.23	0.62
	>200,000	0.18	0.55	0.01	−0.89	1.26	0.74
HL		−0.43	0.06	−0.22	−0.54	−0.31	<0.001
eHL		0.00	0.04	0.00	−0.08	0.08	0.97
PHTC19		1.32	0.16	0.25	0.99	1.64	<0.001
CCBS		0.19	0.07	0.08	0.05	0.33	0.010

Abbreviations: B—unstandardized regression coefficient, SE—standard error, β—standardised regression coefficient, LL—lower limit of 95% confidence interval, UL—upper limit of 95% confidence interval, ref.—category of the variable used as referential for other categories, HL—health literacy, eHL—ehealth literacy, PHTC19—the perceived health threat related to the COVID-19 pandemic; CCBS—COVID-19-related conspiracy belief scale.

**Table 4:** Hierarchical linear regression modelling of FASS.

Variable	Response Options	Model No. 1			Model No. 2			Model No. 3			Model No. 4		
		B (SE)	β	*p*	B (SE)	β	*p*	B (SE)	β	*p*	B (SE)	β	*p*
sex ^1^		−1.43 (0.42)	−0.11	0.001	−1.5 (0.41)	−0.12	<0.001	−1.34 (0.4)	−0.11	0.001	−1.28 (0.39)	−0.10	0.001
income ^2^	1500–3000 PLN	−0.86 (0.52)	−0.07	0.099	−0.86 (0.51)	−0.07	0.089	−0.87 (0.49)	−0.07	0.078	−0.71 (0.49)	−0.05	0.14
	>3000 PLN	−0.86 (0.66)	−0.05	0.19	−0.97 (0.64)	−0.06	0.13	−1.1 (0.62)	−0.07	0.075	−1.00 (0.61)	−0.06	0.10
	refused to reveal	−1.41 (0.72)	−0.07	0.051	−1.45 (0.70)	−0.07	0.039	−1.39 (0.68)	−0.07	0.042	−1.27 (0.67)	−0.06	0.059
vocational status ^3^	entrepreneur or farmer	−1.66 (0.64)	−0.09	0.010	−1.9 (0.63)	−0.1	0.002	−1.62 (0.61)	−0.09	0.008	−1.55 (0.6)	−0.08	0.010
	on a disability pension or retired	−0.62 (0.75)	−0.03	0.41	−0.44 (0.73)	−0.02	0.55	−0.5 (0.71)	−0.02	0.47	−0.32 (0.7)	−0.01	0.64
	University of school student	0.4 (0.76)	0.02	0.60	−0.14 (0.74)	−0.01	0.85	0.4 (0.72)	0.02	0.58	0.64 (0.72)	0.03	0.37
	vocationally inactive incl. unemployed	0.55 (0.58)	0.03	0.34	0.59 (0.56)	0.04	0.29	0.66 (0.54)	0.04	0.22	0.69 (0.54)	0.04	0.20
HL					−0.45 (0.06)	−0.23	<0.001	−0.44 (0.06)	−0.23	<0.001	−0.43 (0.06)	−0.22	<0.001
PHTC19								1.3 (0.16)	0.24	<0.001	1.49 (0.17)	0.28	<0.001
CCBS score											0.33 (0.07)	0.14	<0.001
Raw/Corrected R2		0.031/0.023			0.085/0.077			0.144/0.134			0.162/0.152		
F for change in R2 ^#^		3.791			55.950			63.936			20.750		
ANOVA test for the model-F value *		3.781			9.782			15.785			16.537		

^1^—females as referential category for male, ^2^—monthly net income level per household member equal 1500–3000 PLN as referential category; ^3^—employee of public or private sector as referential category; ^#^—*p* values for the significance of R2 change for all four models <0.001; *—*p* value for ANOVA test for all four models <0.001; Abbreviations: B—unstandardized regression coefficient, SE—standard error, β—standardised regression coefficient, HL—health literacy, eHL—ehealth literacy, PHTC19—the perceived health threat related to the COVID-19 pandemic; CCBS—COVID-19-related conspiracy belief scale.

## Data Availability

The data analysed in this study are available on reasonable request to the corresponding author.
